# Analysis of Retinal Perfusion in Children, Adolescents, and Young Adults with Type 1 Diabetes Using Optical Coherence Tomography Angiography

**DOI:** 10.1155/2019/5410672

**Published:** 2019-05-08

**Authors:** Chiara Mameli, Alessandro Invernizzi, Alice Bolchini, Giorgio Bedogni, Elisa Giani, Maddalena Macedoni, Gianvincenzo Zuccotti, Chiara Preziosa, Marco Pellegrini

**Affiliations:** ^1^Department of Pediatrics, Vittore Buzzi Children's Hospital, Department of Biomedical and Clinical Science, University of Milan, Milan, Italy; ^2^Eye Clinic, Department of Biomedical and Clinical Science, Luigi Sacco Hospital, University of Milan, Milan, Italy; ^3^Save Sight Institute, University of Sydney, Sydney, Australia; ^4^Clinical Epidemiology Unit, Liver Research Center, Basovizza, Trieste, Italy

## Abstract

We performed a cross-sectional study to analyze the retinal vasculature in children, adolescent, and young adults with type 1 diabetes using optical coherence tomography angiography (OCTA). Patients underwent funduscopic examination for diabetic retinopathy (DR) screening during an annual visit for the screening of diabetes-related complications which included the evaluation of glycated hemoglobin (HbA1c), microalbuminuria, lipid profile, arterial pressure, and neurological assessment. In addition, OCTA of the retinal vasculature was performed. Quantitative analysis of the OCTA images evaluated the vessel density at the superficial (SCP) and deep (DCP) capillary plexus of the retina. Structural vascular alterations were evaluated qualitatively. Results were compared to those obtained in a group of healthy age-, sex-, and pubertal stage-matched controls. The effect of age, disease duration, age at the disease onset, mean HbA1c since the onset, and lipid profile on vascular density was tested. Fifty-three patients (median age 15.5, IQR 12.4-19.4 years; 57% females) with type 1 diabetes and 48 controls were enrolled. The median (IQR) HbA1c was 7.6% (60 mmol/mol) (6.9-8.1%, 52-65 mmol/mol), and the median (IQR) duration of disease was 6.0 (3.3-10.3) years. Mean vessel density measured with OCTA was lower in patients compared to controls with the temporal sector showing the highest difference both in the SCP (0.55 vs. 0.57, *p* < 0.001) and the DCP (0.63 vs. 0.65, *p* < 0.001). None of the predictors was associated with the superficial and deep vascular densities. Only 2 patients had clinically detectable DR. Microvascular structural changes were found on OCTA in both of these patients and in one without funduscopic alterations. In conclusion, patients with type 1 diabetes without clinically detectable DR had decreased capillary density compared to controls on OCTA images. These findings may provide useful information for the screening and the management of patients with type 1 diabetes. Further studies are needed to confirm our results and their clinical relevance.

## 1. Introduction

Type 1 diabetes is the most common metabolic disorder of childhood and adolescence. Its onset in pediatric age together with suboptimal metabolic control puts patients at a greater risk of developing diabetes-related complications [1]. Diabetic retinopathy (DR) is one of the most feared complications of diabetes, leading to visual impairment and blindness if untreated. DR is uncommon before puberty, especially in children aged less than 15 years [2]. The reported prevalence of DR in children and young adults ranges from 10% to 58% depending on the studied population [3–7].

The development and progression of DR depend on several modifiable (glucose control, smoking, arterial hypertension, dyslipidemia, and obesity) and nonmodifiable (age, duration of disease, pubertal status, and genetic predisposition) factors [8]. The pathogenesis of DR is far from being completely elucidated. Changes in retinal blood vessel morphology and retinal blood flow have been reported in DR, but little is known about blood flow alterations during preclinical stages. Functional alterations such as increased vascular permeability and leukostasis have been shown to precede pericyte loss and vascular remodeling [9].

The only available imaging technique to evaluate retinal vasculature before 2000s was fluorescein angiography. This is an invasive examination, requiring an intravenous dye injection, with the risk of local and systemic adverse events. Over the last 4 years, a novel noninvasive technique named optical coherence tomography angiography (OCTA) has been introduced in the clinical practice [10]. OCTA provides a noninvasive, rapid, high-resolution assessment of retinal vascular layers with no need for dye injection. Since its introduction in the clinical practice, OCTA has been applied to several eye diseases offering pathogenetic and prognostic insights. A great advantage of OCTA vs. fluorescein angiography is its ability to study separately the different retinal vascular layers and split the superficial (SCP) from deep (DCP) retinal capillary plexus [10].

Recent studies using OCTA have shown that adults affected by diabetes have low capillary density and that adult patients with type 1 diabetes without clinically evident DR have SCP and DCP anomalies [11–15]. To the best of our knowledge, only one study used OCTA to evaluate retinal vessel density in children affected by type 1 diabetes and found no alterations of SCP, DCP, and the fovea avascular zone [16].

The aim of the present cross-sectional study was to use OCTA to evaluate the retinal perfusion of children, adolescents, and young adults with type 1 diabetes and to compare OCTA findings of these patients with a group of age- and sex-matched healthy controls.

## 2. Materials and Methods

### 2.1. Subjects

This cross-sectional study was performed between May 1^st^, 2017, and July 31^st^, 2017, on consecutive patients affected by type 1 diabetes followed up at the Diabetes Clinic of the Vittore Buzzi Children's Hospital (ASST Fatebenefratelli-Sacco, Milan, Italy). The patients were being regularly followed up at our clinic since the diagnosis of type 1 diabetes, and the data for the present study were collected during an annual visit for the screening of diabetes-related complications.

The inclusion criteria were diagnosis of type 1 diabetes, intensive insulin therapy, age between 6 and 25 years, and diabetes duration for at least 6 months. The exclusion criteria were type 2 diabetes mellitus, maturity onset diabetes of the young and syndromic diabetes (e.g., diabetes associated with Down syndrome), myopia exceeding 6.00 diopters, history of nondiabetic retinal disease, previous ocular surgery or laser treatments, ocular media opacities, and poor cooperation. Healthy sex-, pubertal stage-, and age-matched controls were recruited at the Eye Clinic in Luigi Sacco Hospital Milan. The exclusion criteria for controls were myopia exceeding 6.00 diopters, history of any retinal disease, previous ocular surgery or laser treatment, ocular media opacities, and poor cooperation.

Each patient affected by type 1 diabetes underwent a medical, neurological, and comprehensive ophthalmological examination including best-corrected visual acuity (BCVA) assessment, fundus examination, and OCTA as described in detail below [17]. The healthy control underwent BVCA assessment, fundus examination, and OCTA.

All procedures were free of charge and paid by the National Health System.

The study was conducted according to the Declaration of Helsinki and was approved by the local Ethical Committee. Written informed consent was obtained from the subjects aged 18 years or older or from the parents of those younger than 18 years.

### 2.2. Clinical Assessment

The following data were collected for each patient at the time of OCTA: gender, ethnicity, age, height, weight, body mass index (BMI), age at diagnosis of type 1 diabetes, disease duration, type of insulin therapy (multiple daily injections or MDI; continuous subcutaneous insulin infusion or CSII), and insulin requirement (unit/kg/day).

The standard deviation scores (SDS) of weight, height, and BMI were calculated using the World Health Organization (WHO) [18] and the Italian reference data [19]. The pubertal stage was assessed according to Tanner and Whitehouse [20]. Tanner stages 1 and 2 were combined as prepubertal/early pubertal, stages 3 and 4 as pubertal, and stage 5 as postpubertal development.

Systolic blood pressure and diastolic blood pressure were measured following international guidelines [21].

Blood samples were obtained after a 10-hour overnight fast. Creatinine, total cholesterol (TC), high-density lipoprotein cholesterol (HDL), low-density lipoprotein cholesterol (LDL), and triglycerides (TG) were measured using standard laboratory methods. We used standard cut-off values for levels of total cholesterol (hypercholesterolemia ≥ 200 mg per deciliter), HDL cholesterol (hypoHDL < 40 mg per deciliter and <50 mg/dl for females > 16 years), LDL cholesterol (hyperLDL ≥ 130 mg per deciliter), and triglycerides (hyperTG ≥ 150 mg per deciliter) [22].

HbA1c was measured using a fully automated high-performance liquid chromatography system (Variant II, Bio-Rad Laboratories, Munich, Germany).

A first morning urine sample was obtained in all patients to evaluate microalbuminuria.

All samples were analyzed by the laboratory of ASST-Fatebenefratelli Sacco.

The following data were obtained from the medical charts: glycated hemoglobin (HbA1c) at diagnosis and every 3 months, number of hospital admissions for diabetic ketoacidosis (DKA), number of severe hypoglycemic episodes, and presence of diabetes-related complications.

### 2.3. Ophthalmological Assessment

All study subjects underwent a complete ophthalmological examination including BCVA assessment, slit lamp examination, and funduscopic examination performed by a senior ophthalmologist (MP). Pupils were dilated with 1% tropicamide before funduscopic examination and OCTA image collection.

OCTA images were obtained using the split-spectrum amplitude-decorrelation angiography algorithm (SSADA) on the AngioVue OCT-A system version 2017.1.0.15 (Optovue RTVue XR Avanti, Optovue Inc., Freemont, California, USA). This device uses an 840 nm wavelength laser to capture 70,000 A-scans per second; 304 A-scans made up a B-scan, while 304 vertical and horizontal lines were sampled in the scanning area to obtain a 3D data cube. 3 × 3 and 6 × 6 mm volume scans centered onto the fovea were performed in both eyes for each patient.

Automated segmentation of SCP and DCP was performed using the inbuilt software algorithm which sets the inner margin of SCP at 3 *μ*m below the internal limiting membrane of the retina and the outer boundary at 15 *μ*m beneath the inner plexiform layer (IPL), with the DCP top boundary set at 15 *μ*m beneath the IPL and the bottom margin at 71 *μ*m under the IPL.

Vascular density was calculated with the AngioVue Analytics software, which reports the relative density of flow as percentage of the total area. In detail, the vessel density was defined as the percentage of area occupied by vessel lumens after binary reconstruction of images. In the present study, vessel density was calculated for different sectors (superior, nasal, inferior, and temporal) based on the Early Treatment Diabetic Retinopathy Study (ETDRS) chart with the fovea position automatically determined from OCTA. The parafovea was defined as the area within an annulus located between 1 and 2.5 mm from the central fovea.

The SCP and DCP vascular data were analyzed separately. For statistical analysis, we collected the SCP and DCP foveal and parafoveal data from 3 × 3 mm volumetric scans, and the superior, nasal, inferior, and temporal data from 6 × 6 mm images. Data from both eyes of all subjects were considered for analysis (see below).

A qualitative evaluation of the OCTA images was also performed by two double-masked ophthalmologists (MP and CP). For each image, the following DR angiographic features were evaluated on both SPC and DPC OCTA images: presence of microaneurysms, rarefaction of perifoveal capillaries, capillary tortuosity, and disruption of the perifoveolar capillary arcades.

### 2.4. Statistical Analysis

Most continuous variables were not Gaussian-distributed, and all are reported as median and interquartile range (IQR). Discrete variables are reported as the number and proportion of subjects with the characteristic of interest. Between-group comparisons were performed using a fractional generalized linear model (GLM) with a logit link and cluster confidence intervals [23, 24]. The outcome of the fractional GLM was the vascular density (fraction, with theoretical limits from 0 to 1) of the regions of interest (SCP and DCP foveal, parafoveal, temporal, superior, nasal, and inferior areas), and the predictor was type 1 diabetes (discrete: 0 = no; 1 = yes). For each subject, we analyzed both the eyes of each subject in the same model using cluster confidence intervals to take into account the fact that every subject contributed two eyes to the analysis [23]. Because of the 14 multiple comparisons involving SCP and DCP, a significant *p* value was set at *p* < 0.003 (0.05/14). The effect of age, disease duration, age at disease onset, mean HbA1c, LDL, HDL, TG, and TC on vascular density was tested adding each predictor to the fractional GLM.

## 3. Results

### 3.1. Patients

Fifty-five consecutive patients affected by type 1 diabetes were eligible for the study, but two refused to participate. Fifty-three patients with type 1 diabetes were hence included in the analysis. The median (IQR) age was 15.5 (12.4-19.4) years. Thirty (57%) patients were female, and 27 (51%) were being treated with CSII. The median (IQR) HbA1c was 7.6% (60 mmol/mol) (6.9-8.1%, 52-65 mmol/mol), and the median (IQR) duration of disease was 6.0 (3.3-10.3) years. No patient had arterial hypertension, and all patients had a normal neurological evaluation. Two patients had microalbuminuria, i.e., >30 mg/g creatinine/day. The median (IQR) HbA1c from the onset to OCTA was 7.5% (58 mmol/mol) (IQR 7.0-8.0%/53-64 mmol/mol). HyperTC and hyperLDL were detected in 3 patients. Two patients had hyperTG and 6 patients hypoHDL. Detailed data about the 53 patients with type 1 diabetes at the time of OCTA are reported in Table 1.

Forty-eight healthy subjects with a median (IQR) age of 13.7 (11.0-18.9) years served as controls. Forty-five (94%) of them were Caucasians, and 26 (54%) were girls.

### 3.2. Eyes

A total of 106 (53 × 2) eyes from patients with type 1 diabetes and 96 (48 × 2) eyes from healthy controls were analyzed. Tables S1 and S2 report the vascular densities of the SCP and DCP plexus for the right and left eyes of patients with type 1 diabetes and healthy controls. These tables are reported only for descriptive purposes because the comparison between the vascular densities of the SCP and DCP plexus of patients with type 1 diabetes and healthy controls was performed on both eyes using a fractional GLM treating the eyes as clusterized in a given subject (see Statistical Analysis for details).

Vascular density was decreased in patients affected by type 1 diabetes compared to healthy subjects in all the regions of interest in both the superficial and deep plexus with the exception of the fovea. After Bonferroni correction, in the superficial plexus, the mean difference in vascular density between diabetics and controls was significant only in the temporal and the superior regions (0.57 vs. 0.55%, *p* < 0.001 and 0.57 vs. 0.55%, *p* = 0.002, respectively). In the deep plexus, the difference was significant only in the temporal region (0.65 vs. 0.63%, *p* < 0.001).  Table 2 gives the mean (95% confidence intervals (95% CI)) vascular densities for the SCP and DCP in patients with type 1 diabetes and healthy controls estimated by the fractional GLM model described under Statistical Analysis. As shown in Table S3, none of the additional predictors was associated in a clinically relevant way with the superficial and deep vascular densities evaluated by OCTA. (The presence of statistical significance for few predictors was expected by chance alone because of the number of tested models [96] and is clearly irrelevant because the contribution was always negligible on clinical grounds).

**Figure 1:**
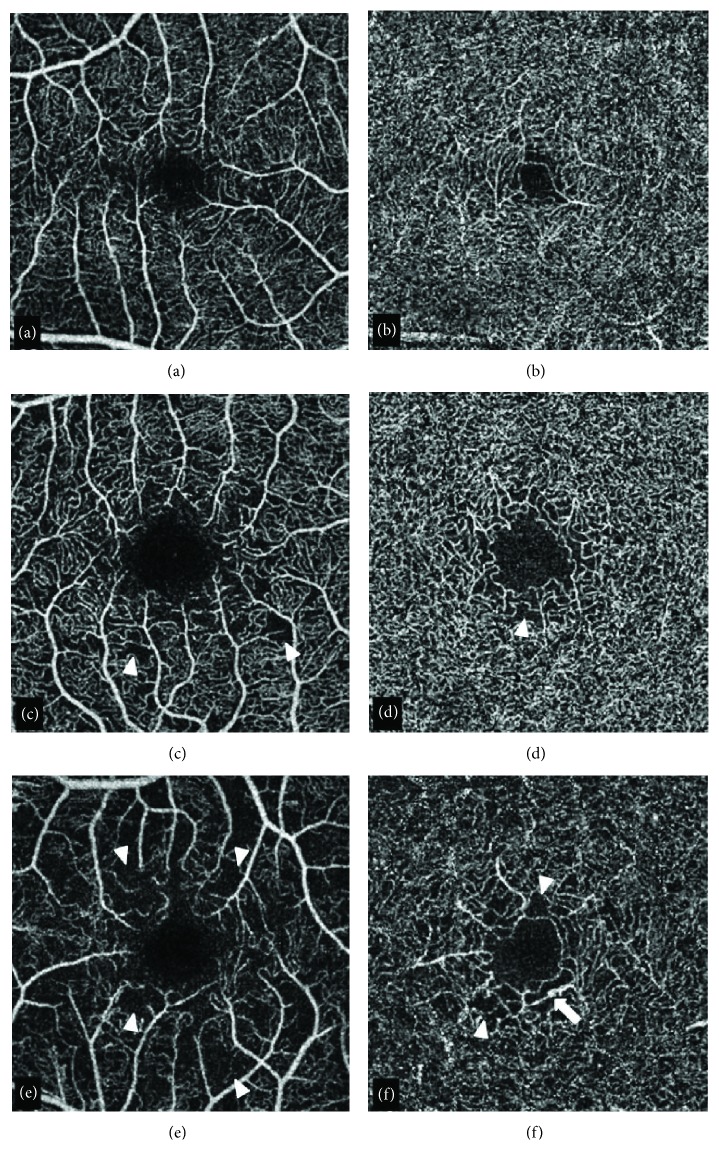
Optical coherence tomography angiography images. (a, b) Optical coherence tomography angiography (OCTA) in a healthy control showing the typical spider web appearance of the superficial capillary plexus (SCP) (a) and the sea fan pattern of the deep capillary plexus (DCP) (b). (c–f) SCP (c and e) and DCP (d and f) in two patients (c and d) patient #40; (e and f) patient #28) showing early vascular changes at the level of both retinal plexus. In these cases, indeed mild capillary loss with reduced vascular density was better visible at the level of the SCP whereas microaneurysms could be recognized at the level of the deep vascular structures. Arrow: microaneurysm; arrowhead: areas of capillary drop-out.

OCTA images were reviewed by 2 masked operators with good agreement, and vascular abnormalities were detected in 3 cases (Figure 1).

## 4. Discussion

DR has long been considered extremely rare in pediatric age. However, a recent large US study has shown an increase of 20% (95% CI 6 to 35%) of the hazard of DR for every 1-point increase of HbA1c in children with type 1 diabetes [25]. Moreover, DR seems to progress rapidly in children with type 1 diabetes, so its early detection can be beneficial [25].

The worldwide increase in the incidence of type 1 diabetes mandates the consideration of strategies for the early detection of DR. OCTA may be one of such strategies as studies of diabetic adults have shown that OCTA can detect alterations of retinal blood flow in the presence of normal fundus examinations [14, 15].

In the present study, we found that OCTA was able to detect significantly lower retinal vessel density in temporal SCP (0.55 vs. 0.57) and DCP (0.63 vs. 0.65) in both eyes of patients with type 1 diabetes mostly with no clinically detectable DR compared to healthy children. Reduced vessel density was also detected in the other regions of interest of both SCP and DCP of the same eyes except for the foveal region. This partially confirms recent studies reporting vascular changes in the DCP of patients affected by type 1 diabetes [12, 13].

Our results are intriguing since they demonstrate that OCTA can identify early microvascular changes that occur in the retina before the DR becomes clinically visible and that are consequently not detectable with the standard screening procedures. However, the clinical significance of the differences we found in capillary density between patients affected by type 1 diabetes and controls is unclear because of their size even when statistically significant. For instance, the mean (SD) difference estimated by the fractional GLM for S-Superior is -0.02 (95% CI -0.03 to -0.01) for patients with type 1 diabetes *vs.* healthy subjects. Although statistically significant, a reduction of 0.02 is about 4% of the density (0.57) of the control group. Nevertheless, it is interesting to note that in our analysis, the temporal macular sectors resulted to be primarily affected in both the SCP and DCP. This is in agreement with previous findings from other researches evidencing this sector as a particularly susceptible portion of the macula [26]. Han et al. in 2017 described OCTA changes in patients affected by various sickle cell genotypes describing greater flow loss in the temporal subfields [26]. One of the main explanations for this finding was that the temporal macular vessels are located within the watershed zones along the horizontal raphe and consequently nearby terminal vessels. This anatomical configuration may lead to increase susceptibility of vascular damage for this sector. Also, these results confirm previous researches showing retinal thinning to be more prominent in the temporal sectors in the same subset of patients.

Our results could suggest that in a cohort of well-controlled children and young adults with short duration of disease, alterations in retinal vessel density occur very early and before the onset of other diabetes-related complications. We could speculate that retina is one of the most sensitive target tissues. The lack of correlation with median HbA1c value since the disease onset could suggest that we should try to look over hemoglobin A1C and search for new metrics in the clinical practice to estimate the risk of diabetes-related chronic complications. In this field, the role “time in range” is emergent. In adults affected by type 2 diabetes, the lowest percentage of time in range is associated with an increased risk to have diabetic retinopathy and an increased severity of eye damage [27, 28].

The prevalence of clinically detectable DR was very low in our population, i.e., 3‰ (2/53 patients). The low frequency and the mild form in our population of DR are not surprising owing to the low median duration of disease of our patients (6 years). It must also be pointed out that the median (IQR) HbA1c at the time of OCTA was 7.6% (60 mmol/mol) (6.9-8.1; 52-65 mmol/mol), which is compatible with a reasonably good metabolic control in the last three months.

In our series, we performed both a qualitative and a quantitative assessment of retinal vascular alterations occurring in both populations. OCTA qualitative analysis showed vascular abnormalities (microaneurysms, remodeling, and capillary loss) in patients that had clinically detectable DR. Similar qualitative microvascular abnormalities were also detected on OCTA in a patient with long duration of disease and suboptimal metabolic control since diagnosis but with a normal fundus. Interestingly, all the three patients with OCTA-detected retinal alterations had disease duration above the median, i.e., 8, 11, and 20 years.

Even if this is one of the first studies to investigate the role of OCTA in detecting early alterations of the macular capillary network in pediatric patients with type 1 diabetes, it has several limitations. First, this is a cross-sectional study, and as such, it can evaluate the retinal vasculature status only at a specific time point. A prospective evaluation of these children will help us to describe the evolution of our findings. Second, the population recruited is relatively small. The main reason is the difficultly in recruiting children especially in prepubertal age because OCTA requires patient's collaboration. However, considering the available studies published so far on the use of OCTA in pediatric population affected by type 1 diabetes without diabetic retinopathy, our cohort was one of the largest.

Third, recent updates in OCTA software are looking for the possibility to further split the DCP in two separate vascular structures, and the recently described intermediate plexus may be primarily affected in the very early phases of the disease.

## 5. Conclusions

This study investigates the role of OCTA in detecting early vascular changes in pediatric patients with type 1 diabetes. OCTA is confirmed to be a valuable tool for noninvasive diagnosing and monitoring of these patients. In particular, we found that microvascular changes in both SCP and DCP could be reliably highlighted by means of OCTA before the appearance of clinical signs of DR at fundoscopy. In our series, the temporal sectors appeared to be more significantly reduced compared to the other subfields thus possibly representing a primary site of pathology. Cohort studies are needed to further evaluate the potential of OCTA for the screening of DR in children with type 1 diabetes, for improving the treatment of diabetes-related ocular disease and to understand the relevance of intraocular early signs in the progression of the systemic disease.

## Figures and Tables

**Table 1:** Patient characteristics.

	Patient with type 1 diabetes		Healthy controls	
	*N* (%)	Median (IQR)	*N* (%)	Median (IQR)
Gender	53		48	
Female	30 (57%)		26 (54%)	
Male	23 (43%)		22 (46%)	
Age (years)	53	15.5 (12.4; 19.4)	48	13.7 (11.8-18.9)
Caucasian	53		48	
No	7 (13%)		3 (6%)	
Yes	46 (87%)		45 (94%)	
Weight (kg)	52	59 (45; 65)		
Height (m)	52	1.62 (1.54; 1.71)		
BMI (kg/m^2^)	52	21.4 (19.3; 23.8)		
Weight (SDS Cacciari)	38	0.02 (-0.54; 0.86)		
Height (SDS Cacciari)	38	0.23 (-0.41; 0.95)		
BMI (SDS Cacciari)	38	-0.08 (-0.51; 0.58)		
Prepubertal	10		10	
Pubertal	12		12	
Postpubertal	31		26	
Diabetes duration (years)	53	6.0 (3.3; 10.3)		
Insulin treatment	53			
Multiple daily injections	26 (49%)			
CSII	27 (51%)			
HbA1c (%)	53	7.6 (6.9; 8.1)		
HbA1c (mmol/mol)	53	60 (52; 65)		
Microalbuminuria (mg/g creatinine/day)	43	6.0 (5.0; 10.0)		
Cholesterol (mg/dl)	53	169 (151; 182)		
HDL cholesterol (mg/dl)	53	51 (45; 60)		
LDL cholesterol (mg/dl)	53	102 (80; 115)		
Triglycerides (mg/dl)	53	58 (47; 75)		

Data are reported as median (interquartile range) for continuous measures and *n* (%) for categorical measure. BMI = body mass index; Cacciari = Italian reference data; CSII: continuous subcutaneous insulin infusion; HbA1c = glycated hemoglobin; HDL = high-density lipoprotein; IQR = interquartile range; LDL = low-density lipoprotein; SDS = standard deviation score.

**Table 2:** Vascular densities of the superficial and deep plexus in patients with type 1 diabetes and healthy controls.

	Healthy controls (*n* = 96 eyes)			Patients with type 1 diabetes (*n* = 106 eyes)			*p* value
	Mean	95% LCI	95% UCI	Mean	95% LCI	95% UCI	
S-Fovea	0.31	0.30	0.32	0.32	0.31	0.33	0.3025
S-Parafovea	0.58	0.57	0.58	0.57	0.56	0.57	0.0054
S-Temporal	0.57	0.56	0.57	0.55	0.54	0.55	<0.001
S-Superior	0.57	0.56	0.58	0.55	0.54	0.56	0.0028
S-Nasal	0.57	0.56	0.58	0.56	0.55	0.56	0.0068
S-Inferior	0.56	0.55	0.57	0.55	0.54	0.56	0.0229
D-Fovea	0.31	0.29	0.32	0.33	0.31	0.35	0.0404
D-Parafovea	0.65	0.65	0.65	0.64	0.64	0.65	0.0036
D-Temporal	0.65	0.64	0.65	0.63	0.63	0.64	<0.001
D-Superior	0.66	0.65	0.66	0.65	0.65	0.66	0.7267
D-Nasal	0.65	0.65	0.66	0.65	0.64	0.65	0.5897
D-Inferior	0.65	0.65	0.66	0.65	0.65	0.66	0.8462

Comparison of the vascular densities of the superficial and deep plexus in patients with type 1 diabetes and healthy controls. Values are means and 95% confidence intervals estimated from a fractional generalized linear model (see Statistical Analysis for details).LCI = lower confidence interval; UCI = upper confidence interval; S- = superficial plexus; D- = deep plexus.

## Data Availability

The data used to support the findings of this study are available from the corresponding author upon request.

## Supplementary Materials

Supplementary Materials Tables S1 and S2 report the vascular densities of the SCP and DCP plexus for the right and left eyes of patients with type 1 diabetes and healthy controls. Table S3 shows the correlations between predictors and the superficial and deep vascular densities.
