# Supplementary material for: Analysis of Retinal Perfusion in Children, Adolescents, and Young Adults with Type 1 Diabetes Using Optical Coherence Tomography Angiography
Source: J Diabetes Res. 2019 May 8;2019:5410672. doi: 10.1155/2019/5410672 (PMC6530197; doi:10.1155/2019/5410672)
Supplement: Supplementary Materials — Tables S1 and S2 report the vascular densities of the SCP and DCP plexus for the right and left eyes of patients with type 1 diabetes and healthy controls. Table S3 shows the correlations between predictors and the superficial and deep vascular densities. [file 5410672.f1.pdf]

Table S1. Vascular densities of the right eye in patients with type 1 diabetes and healthy controls

	Controls<br>N = 48	Diabetes type 1<br>N = 53
S-Fovea	0.32 (0.28; 0.34)	0.31 (0.29; 0.35)
S-ParaFovea	0.58 (0.57; 0.59)	0.57 (0.55; 0.58)
S-Temporal	0.57 (0.55; 0.59)	0.55 (0.55; 0.56)
S-Superior	0.57 (0.54; 0.59)	0.56 (0.54; 0.58)
S-Nasal	0.57 (0.55; 0.60)	0.57 (0.55; 0.58)
S-Inferior	0.57 (0.54; 0.59)	0.56 (0.53; 0.57)
D-Fovea	0.30 (0.27; 0.36)	0.33 (0.29; 0.37)
D-ParaFovea	0.65 (0.64; 0.66)	0.64 (0.63; 0.65)
D-Temporal	0.65 (0.64; 0.66)	0.64 (0.62; 0.65)
D-Superior	0.66 (0.64; 0.67)	0.66 (0.64; 0.67)
D-Nasal	0.65 (0.64; 0.67)	0.65 (0.64; 0.66)
D-Inferior	0.66 (0.64; 0.68)	0.66 (0.64; 0.67)

Legend to table S1

Values are medians and interquartile ranges and are provided for descriptive purposes only. Abbreviations: S- = superficial plexus; D- = deep plexus.

Table S2. **Vascular densities of the left eye in patients with type 1 diabetes and healthy controls**

	Controls<br>N = 48	Type 1 diabetes<br>N = 53
S-Fovea	0.31 (0.27; 0.34)	0.32 (0.28; 0.35)
S-ParaFovea	0.58 (0.56; 0.59)	0.57 (0.56; 0.58)
S-Temporal	0.57 (0.55; 0.58)	0.55 (0.54; 0.55)
S-Superior	0.57 (0.56; 0.59)	0.56 (0.54; 0.58)
S-Nasal	0.57 (0.56; 0.59)	0.56 (0.54; 0.58)
S-Inferior	0.56 (0.54; 0.58)	0.55 (0.53; 0.57)
D-Fovea	0.31 (0.26; 0.34)	0.32 (0.29; 0.37)
D-ParaFovea	0.65 (0.64; 0.66)	0.65 (0.64; 0.66)
D-Temporal	0.64 (0.63; 0.67)	0.63 (0.61; 0.65)
D-Superior	0.66 (0.64; 0.67)	0.65 (0.64; 0.67)
D-Nasal	0.65 (0.64; 0.66)	0.65 (0.64; 0.66)
D-Inferior	0.66 (0.64; 0.67)	0.66 (0.64; 0.67)

Legend to table S2

Values are medians and interquartile ranges and are provided for descriptive purposes only.

Abbreviations: S- = superficial plexus; D- = deep plexus.

Table S3. Predictors and vascular densities in patients with type 1 diabetes and healthy controls

	M1
SFovea	
Age (years)	0.007
	[-0.002,0.016]
T1DM	0.036
	[-0.044,0.116]
Constant	-0.901***
	[-1.049,-0.753]
Observations	202

95% confidence intervals in brackets
Values are regression coefficients from fractional GLM with logit link
* $p < 0.05$, ** $p < 0.01$, *** $p < 0.001$

	M2
SFovea	
T1DM duration (years)	-0.003
	[-0.015,0.009]
T1DM	0.000
	[0.000,0.000]
Constant	-0.738***
	[-0.835,-0.640]
Observations	106

95% confidence intervals in brackets
Values are regression coefficients from fractional GLM with logit link
* $p < 0.05$, ** $p < 0.01$, *** $p < 0.001$

	M3
SFovea	
Age at onset (years)	0.006
	[-0.009,0.020]
T1DM	0.000
	[0.000,0.000]
Constant	-0.809***
	[-0.958,-0.659]
Observations	106

95% confidence intervals in brackets
Values are regression coefficients from fractional GLM with logit link
* $p < 0.05$, ** $p < 0.01$, *** $p < 0.001$

	M4
SFovea	
Mean HbA1c from	-0.019

onset to OCT (%)	[-0.050,0.011]
T1DM	0.000
	[0.000,0.000]
Constant	-0.615***
	[-0.865,-0.365]
Observations	100

95% confidence intervals in brackets
Values are regression coefficients from fractional GLM with logit link
* $p < 0.05$, ** $p < 0.01$, *** $p < 0.001$

	M5
SFovea	
LDL-cholesterol (mg/dl)	0.002
	[-0.001,0.005]
T1DM	0.000
	[0.000,0.000]
Constant	-0.956***
	[-1.227,-0.684]
Observations	106

95% confidence intervals in brackets
Values are regression coefficients from fractional GLM with logit link
* $p < 0.05$, ** $p < 0.01$, *** $p < 0.001$

	M6
SFovea	
HDL-cholesterol (mg/dl)	0.005*
	[0.000,0.010]
T1DM	0.000
	[0.000,0.000]
Constant	-1.023***
	[-1.271,-0.775]
Observations	106

95% confidence intervals in brackets
Values are regression coefficients from fractional GLM with logit link
* $p < 0.05$, ** $p < 0.01$, *** $p < 0.001$

	M7
SFovea	
Cholesterol (mg/dl)	0.002*
	[0.000,0.004]
T1DM	0.000
	[0.000,0.000]
Constant	-1.116***
	[-1.455,-0.776]

Observations	106

95% confidence intervals in brackets
Values are regression coefficients from fractional GLM with logit link
* $p < 0.05$, ** $p < 0.01$, *** $p < 0.001$

	M8
SFovea	
Triglycerides (mg/dl)	-0.000
	[-0.001,0.001]
T1DM	0.000
	[0.000,0.000]
Constant	-0.750***
	[-0.856,-0.645]
Observations	106

95% confidence intervals in brackets
Values are regression coefficients from fractional GLM with logit link
* $p < 0.05$, ** $p < 0.01$, *** $p < 0.001$

	M9
SParaFovea	
Age (years)	0.001
	[-0.002,0.003]
T1DM	-0.049**
	[-0.083,-0.015]
Constant	0.310***
	[0.264,0.356]
Observations	202

95% confidence intervals in brackets
Values are regression coefficients from fractional GLM with logit link
* $p < 0.05$, ** $p < 0.01$, *** $p < 0.001$

	M10
SParaFovea	
T1DM duration (years)	-0.002
	[-0.006,0.002]
T1DM	0.000
	[0.000,0.000]
Constant	0.285***
	[0.246,0.324]
Observations	106

95% confidence intervals in brackets
Values are regression coefficients from fractional GLM with logit link
* $p < 0.05$, ** $p < 0.01$, *** $p < 0.001$

	M11
SParaFovea	
Age at onset (years)	0.004

	[-0.002,0.010]
T1DM	0.000
	[0.000,0.000]
Constant	0.237***
	[0.180,0.293]
Observations	106

95% confidence intervals in brackets
Values are regression coefficients from fractional GLM with logit link
* $p < 0.05$, ** $p < 0.01$, *** $p < 0.001$

	M12
SParaFovea	
Mean HbA1c from onset to OCT (%)	-0.042
	[-0.097,0.014]
T1DM	0.000
	[0.000,0.000]
Constant	0.591**
	[0.178,1.005]
Observations	100

95% confidence intervals in brackets
Values are regression coefficients from fractional GLM with logit link
* $p < 0.05$, ** $p < 0.01$, *** $p < 0.001$

	M13
SParaFovea	
LDL-cholesterol (mg/dl)	0.000
	[-0.001,0.001]
T1DM	0.000
	[0.000,0.000]
Constant	0.256***
	[0.135,0.376]
Observations	106

95% confidence intervals in brackets
Values are regression coefficients from fractional GLM with logit link
* $p < 0.05$, ** $p < 0.01$, *** $p < 0.001$

	M14
SParaFovea	
HDL-cholesterol (mg/dl)	-0.000
	[-0.003,0.002]
T1DM	0.000
	[0.000,0.000]
Constant	0.294***
	[0.190,0.397]

Observations	106

95% confidence intervals in brackets
Values are regression coefficients from fractional GLM with logit link
* $p < 0.05$, ** $p < 0.01$, *** $p < 0.001$

	M15
SParaFovea	
Cholesterol (mg/dl)	-0.000
	[-0.001,0.001]
T1DM	0.000
	[0.000,0.000]
Constant	0.279*
	[0.065,0.492]
Observations	106

95% confidence intervals in brackets
Values are regression coefficients from fractional GLM with logit link
* $p < 0.05$, ** $p < 0.01$, *** $p < 0.001$

	M16
SParaFovea	
Triglycerides (mg/dl)	-0.000
	[-0.001,0.001]
T1DM	0.000
	[0.000,0.000]
Constant	0.282***
	[0.229,0.335]
Observations	106

95% confidence intervals in brackets
Values are regression coefficients from fractional GLM with logit link
* $p < 0.05$, ** $p < 0.01$, *** $p < 0.001$

	M17
STemporal	
Age (years)	0.000
	[-0.003,0.004]
T1DM	-0.077***
	[-0.110,-0.044]
Constant	0.266***
	[0.216,0.316]
Observations	202

95% confidence intervals in brackets
Values are regression coefficients from fractional GLM with logit link
* $p < 0.05$, ** $p < 0.01$, *** $p < 0.001$

	M18
STemporal	
T1DM duration	0.002

(years)	[-0.001,0.005]
T1DM	0.000
	[0.000,0.000]
Constant	0.182***
	[0.153,0.210]
Observations	106

95% confidence intervals in brackets
Values are regression coefficients from fractional GLM with logit link
* $p < 0.05$, ** $p < 0.01$, *** $p < 0.001$

	M19
STemporal	
Age at onset (years)	-0.001
	[-0.006,0.004]
T1DM	0.000
	[0.000,0.000]
Constant	0.205***
	[0.156,0.253]
Observations	106

95% confidence intervals in brackets
Values are regression coefficients from fractional GLM with logit link
* $p < 0.05$, ** $p < 0.01$, *** $p < 0.001$

	M20
STemporal	
Mean HbA1c from onset to OCT (%)	-0.016
	[-0.050,0.019]
T1DM	0.000
	[0.000,0.000]
Constant	0.315*
	[0.059,0.571]
Observations	100

95% confidence intervals in brackets
Values are regression coefficients from fractional GLM with logit link
* $p < 0.05$, ** $p < 0.01$, *** $p < 0.001$

	M21
STemporal	
LDL-cholesterol (mg/dl)	0.000
	[-0.001,0.001]
T1DM	0.000
	[0.000,0.000]
Constant	0.189***
	[0.100,0.279]

Observations	106

95% confidence intervals in brackets
Values are regression coefficients from fractional GLM with logit link
* p < 0.05, ** p < 0.01, *** p < 0.001

	M22
STemporal	
HDL-cholesterol	-0.001
(mg/dl)	[-0.003,0.000]
T1DM	0.000
	[0.000,0.000]
Constant	0.262***
	[0.183,0.342]
Observations	106

95% confidence intervals in brackets
Values are regression coefficients from fractional GLM with logit link
* p < 0.05, ** p < 0.01, *** p < 0.001

	M23
STemporal	
Cholesterol (mg/dl)	-0.000
	[-0.001,0.001]
T1DM	0.000
	[0.000,0.000]
Constant	0.212**
	[0.074,0.350]
Observations	106

95% confidence intervals in brackets
Values are regression coefficients from fractional GLM with logit link
* p < 0.05, ** p < 0.01, *** p < 0.001

	M24
STemporal	
Triglycerides (mg/dl)	0.000
	[-0.000,0.001]
T1DM	0.000
	[0.000,0.000]
Constant	0.179***
	[0.145,0.214]
Observations	106

95% confidence intervals in brackets
Values are regression coefficients from fractional GLM with logit link
* p < 0.05, ** p < 0.01, *** p < 0.001

	M25
SSuperior	
Age (years)	0.004

	[-0.000,0.008]
T1DM	-0.072**
	[-0.115,-0.028]
Constant	0.220***
	[0.154,0.286]
Observations	202

95% confidence intervals in brackets
Values are regression coefficients from fractional GLM with logit link
* $p < 0.05$, ** $p < 0.01$, *** $p < 0.001$

	M26
SSuperior	
T1DM duration (years)	0.002
	[-0.004,0.008]
T1DM	0.000
	[0.000,0.000]
Constant	0.195***
	[0.145,0.245]
Observations	106

95% confidence intervals in brackets
Values are regression coefficients from fractional GLM with logit link
* $p < 0.05$, ** $p < 0.01$, *** $p < 0.001$

	M27
SSuperior	
Age at onset (years)	0.002
	[-0.007,0.010]
T1DM	0.000
	[0.000,0.000]
Constant	0.199***
	[0.120,0.277]
Observations	106

95% confidence intervals in brackets
Values are regression coefficients from fractional GLM with logit link
* $p < 0.05$, ** $p < 0.01$, *** $p < 0.001$

	M28
SSuperior	
Mean HbA1c from onset to OCT (%)	-0.030
	[-0.068,0.008]
T1DM	0.000
	[0.000,0.000]
Constant	0.448**
	[0.164,0.731]

Observations	100

95% confidence intervals in brackets
Values are regression coefficients from fractional GLM with logit link
* $p < 0.05$, ** $p < 0.01$, *** $p < 0.001$

	M29
SSuperior LDL-cholesterol (mg/dl)	0.001
	[-0.001,0.002]
T1DM	0.000
	[0.000,0.000]
Constant	0.161*
	[0.005,0.317]
Observations	106

95% confidence intervals in brackets
Values are regression coefficients from fractional GLM with logit link
* $p < 0.05$, ** $p < 0.01$, *** $p < 0.001$

	M30
SSuperior HDL-cholesterol (mg/dl)	-0.001
	[-0.004,0.001]
T1DM	0.000
	[0.000,0.000]
Constant	0.288***
	[0.147,0.429]
Observations	106

95% confidence intervals in brackets
Values are regression coefficients from fractional GLM with logit link
* $p < 0.05$, ** $p < 0.01$, *** $p < 0.001$

	M31
SSuperior Cholesterol (mg/dl)	0.000
	[-0.001,0.002]
T1DM	0.000
	[0.000,0.000]
Constant	0.171
	[-0.049,0.391]
Observations	106

95% confidence intervals in brackets
Values are regression coefficients from fractional GLM with logit link
* $p < 0.05$, ** $p < 0.01$, *** $p < 0.001$

	M32
SSuperior Triglycerides (mg/dl)	0.000

	[-0.001,0.001]
T1DM	0.000
	[0.000,0.000]
Constant	0.185***
	[0.111,0.260]
Observations	106

95% confidence intervals in brackets
Values are regression coefficients from fractional GLM with logit link
* $p < 0.05$, ** $p < 0.01$, *** $p < 0.001$

	M33
SNasal	
Age (years)	0.001
	[-0.002,0.005]
T1DM	-0.055**
	[-0.093,-0.016]
Constant	0.262***
	[0.210,0.315]
Observations	202

95% confidence intervals in brackets
Values are regression coefficients from fractional GLM with logit link
* $p < 0.05$, ** $p < 0.01$, *** $p < 0.001$

	M34
SNasal	
T1DM duration (years)	0.004
	[-0.001,0.009]
T1DM	0.000
	[0.000,0.000]
Constant	0.202***
	[0.152,0.252]
Observations	106

95% confidence intervals in brackets
Values are regression coefficients from fractional GLM with logit link
* $p < 0.05$, ** $p < 0.01$, *** $p < 0.001$

	M35
SNasal	
Age at onset (years)	-0.004
	[-0.011,0.002]
T1DM	0.000
	[0.000,0.000]
Constant	0.267***
	[0.210,0.323]

Observations	106

95% confidence intervals in brackets
Values are regression coefficients from fractional GLM with logit link
* $p < 0.05$, ** $p < 0.01$, *** $p < 0.001$

	M36
SNasal	
Mean HbA1c from onset to OCT (%)	-0.022
	[-0.057,0.014]
T1DM	0.000
	[0.000,0.000]
Constant	0.399**
	[0.126,0.672]
Observations	100

95% confidence intervals in brackets
Values are regression coefficients from fractional GLM with logit link
* $p < 0.05$, ** $p < 0.01$, *** $p < 0.001$

	M37
SNasal	
LDL-cholesterol (mg/dl)	0.000
	[-0.001,0.002]
T1DM	0.000
	[0.000,0.000]
Constant	0.194**
	[0.047,0.341]
Observations	106

95% confidence intervals in brackets
Values are regression coefficients from fractional GLM with logit link
* $p < 0.05$, ** $p < 0.01$, *** $p < 0.001$

	M38
SNasal	
HDL-cholesterol (mg/dl)	-0.002
	[-0.005,0.000]
T1DM	0.000
	[0.000,0.000]
Constant	0.344***
	[0.208,0.481]
Observations	106

95% confidence intervals in brackets
Values are regression coefficients from fractional GLM with logit link
* $p < 0.05$, ** $p < 0.01$, *** $p < 0.001$

	M39
SNasal	
Cholesterol (mg/dl)	-0.000

	[-0.001,0.001]
T1DM	0.000
	[0.000,0.000]
Constant	0.234*
	[0.014,0.455]
Observations	106

95% confidence intervals in brackets
Values are regression coefficients from fractional GLM with logit link
* $p < 0.05$, ** $p < 0.01$, *** $p < 0.001$

	M40
SNasal	
Triglycerides (mg/dl)	0.000
	[-0.000,0.001]
T1DM	0.000
	[0.000,0.000]
Constant	0.209***
	[0.153,0.266]
Observations	106

95% confidence intervals in brackets
Values are regression coefficients from fractional GLM with logit link
* $p < 0.05$, ** $p < 0.01$, *** $p < 0.001$

	M41
SInferior	
Age (years)	0.003
	[-0.001,0.006]
T1DM	-0.051*
	[-0.091,-0.010]
Constant	0.208***
	[0.147,0.269]
Observations	202

95% confidence intervals in brackets
Values are regression coefficients from fractional GLM with logit link
* $p < 0.05$, ** $p < 0.01$, *** $p < 0.001$

	M42
SInferior	
T1DM duration (years)	-0.000
	[-0.006,0.006]
T1DM	0.000
	[0.000,0.000]
Constant	0.199***
	[0.150,0.248]

Observations	106

95% confidence intervals in brackets
Values are regression coefficients from fractional GLM with logit link
* $p < 0.05$, ** $p < 0.01$, *** $p < 0.001$

	M43
SInferior	
Age at onset (years)	0.002
	[-0.005,0.008]
T1DM	0.000
	[0.000,0.000]
Constant	0.183***
	[0.119,0.247]
Observations	106

95% confidence intervals in brackets
Values are regression coefficients from fractional GLM with logit link
* $p < 0.05$, ** $p < 0.01$, *** $p < 0.001$

	M44
SInferior	
Mean HbA1c from onset to OCT (%)	-0.027
	[-0.076,0.022]
T1DM	0.000
	[0.000,0.000]
Constant	0.406*
	[0.041,0.771]
Observations	100

95% confidence intervals in brackets
Values are regression coefficients from fractional GLM with logit link
* $p < 0.05$, ** $p < 0.01$, *** $p < 0.001$

	M45
SInferior	
LDL-cholesterol (mg/dl)	0.000
	[-0.001,0.002]
T1DM	0.000
	[0.000,0.000]
Constant	0.182*
	[0.038,0.326]
Observations	106

95% confidence intervals in brackets
Values are regression coefficients from fractional GLM with logit link
* $p < 0.05$, ** $p < 0.01$, *** $p < 0.001$

	M46
SInferior	
HDL-cholesterol	-0.002

(mg/dl)	[-0.004,0.001]
T1DM	0.000
	[0.000,0.000]
Constant	0.294***
	[0.155,0.433]
Observations	106

95% confidence intervals in brackets
Values are regression coefficients from fractional GLM with logit link
* $p < 0.05$, ** $p < 0.01$, *** $p < 0.001$

	M47
SInferior	
Cholesterol (mg/dl)	0.000
	[-0.001,0.001]
T1DM	0.000
	[0.000,0.000]
Constant	0.197
	[-0.025,0.419]
Observations	106

95% confidence intervals in brackets
Values are regression coefficients from fractional GLM with logit link
* $p < 0.05$, ** $p < 0.01$, *** $p < 0.001$

	M48
SInferior	
Triglycerides (mg/dl)	0.001
	[-0.000,0.002]
T1DM	0.000
	[0.000,0.000]
Constant	0.160***
	[0.093,0.227]
Observations	106

95% confidence intervals in brackets
Values are regression coefficients from fractional GLM with logit link
* $p < 0.05$, ** $p < 0.01$, *** $p < 0.001$

	M49
DFovea	
Age (years)	0.009
	[-0.003,0.020]
T1DM	0.099
	[-0.005,0.204]
Constant	-0.949***
	[-1.129,-0.769]

Observations	202

95% confidence intervals in brackets
Values are regression coefficients from fractional GLM with logit link
[*] $p < 0.05$, [**] $p < 0.01$, [***] $p < 0.001$

	M50
DFovea	
T1DM duration (years)	0.001
	[-0.013,0.015]
T1DM	0.000
	[0.000,0.000]
Constant	-0.716***
	[-0.848,-0.584]
Observations	106

95% confidence intervals in brackets
Values are regression coefficients from fractional GLM with logit link
[*] $p < 0.05$, [**] $p < 0.01$, [***] $p < 0.001$

	M51
DFovea	
Age at onset (years)	0.005
	[-0.015,0.025]
T1DM	0.000
	[0.000,0.000]
Constant	-0.753***
	[-0.956,-0.550]
Observations	106

95% confidence intervals in brackets
Values are regression coefficients from fractional GLM with logit link
[*] $p < 0.05$, [**] $p < 0.01$, [***] $p < 0.001$

	M52
DFovea	
Mean HbA1c from onset to OCT (%)	-0.002
	[-0.053,0.049]
T1DM	0.000
	[0.000,0.000]
Constant	-0.695***
	[-1.109,-0.281]
Observations	100

95% confidence intervals in brackets
Values are regression coefficients from fractional GLM with logit link
[*] $p < 0.05$, [**] $p < 0.01$, [***] $p < 0.001$

	M53
DFovea	
LDL-cholesterol	0.002

(mg/dl)	[-0.002,0.006]
T1DM	0.000
	[0.000,0.000]
Constant	-0.937***
	[-1.331,-0.543]
Observations	106

95% confidence intervals in brackets
Values are regression coefficients from fractional GLM with logit link
* $p < 0.05$, ** $p < 0.01$, *** $p < 0.001$

	M54
DFovea	
HDL-cholesterol (mg/dl)	0.005
	[-0.002,0.012]
T1DM	0.000
	[0.000,0.000]
Constant	-0.971***
	[-1.331,-0.610]
Observations	106

95% confidence intervals in brackets
Values are regression coefficients from fractional GLM with logit link
* $p < 0.05$, ** $p < 0.01$, *** $p < 0.001$

	M55
DFovea	
Cholesterol (mg/dl)	0.002
	[-0.001,0.006]
T1DM	0.000
	[0.000,0.000]
Constant	-1.106***
	[-1.641,-0.571]
Observations	106

95% confidence intervals in brackets
Values are regression coefficients from fractional GLM with logit link
* $p < 0.05$, ** $p < 0.01$, *** $p < 0.001$

	M56
DFovea	
Triglycerides (mg/dl)	-0.000
	[-0.002,0.002]
T1DM	0.000
	[0.000,0.000]
Constant	-0.710***
	[-0.861,-0.558]

Observations	106

95% confidence intervals in brackets
Values are regression coefficients from fractional GLM with logit link
* $p < 0.05$, ** $p < 0.01$, *** $p < 0.001$

	M57
DParaFovea	
Age (years)	-0.000
	[-0.003,0.002]
T1DM	-0.037**
	[-0.063,-0.012]
Constant	0.626***
	[0.590,0.662]
Observations	202

95% confidence intervals in brackets
Values are regression coefficients from fractional GLM with logit link
* $p < 0.05$, ** $p < 0.01$, *** $p < 0.001$

	M58
DParaFovea	
T1DM duration	-0.003
(years)	[-0.007,0.000]
T1DM	0.000
	[0.000,0.000]
Constant	0.608***
	[0.580,0.636]
Observations	106

95% confidence intervals in brackets
Values are regression coefficients from fractional GLM with logit link
* $p < 0.05$, ** $p < 0.01$, *** $p < 0.001$

	M59
DParaFovea	
Age at onset (years)	0.005*
	[0.001,0.009]
T1DM	0.000
	[0.000,0.000]
Constant	0.541***
	[0.494,0.587]
Observations	106

95% confidence intervals in brackets
Values are regression coefficients from fractional GLM with logit link
* $p < 0.05$, ** $p < 0.01$, *** $p < 0.001$

	M60
DParaFovea	
Mean HbA1c from	-0.040**

onset to OCT (%)	[-0.070,-0.010]
T1DM	0.000
	[0.000,0.000]
Constant	0.892***
	[0.666,1.118]
Observations	100

95% confidence intervals in brackets
Values are regression coefficients from fractional GLM with logit link
* $p < 0.05$, ** $p < 0.01$, *** $p < 0.001$

	M61
DParaFovea	
LDL-cholesterol	0.000
(mg/dl)	[-0.001,0.001]
T1DM	0.000
	[0.000,0.000]
Constant	0.548***
	[0.458,0.639]
Observations	106

95% confidence intervals in brackets
Values are regression coefficients from fractional GLM with logit link
* $p < 0.05$, ** $p < 0.01$, *** $p < 0.001$

	M62
DParaFovea	
HDL-cholesterol	0.000
(mg/dl)	[-0.001,0.001]
T1DM	0.000
	[0.000,0.000]
Constant	0.580***
	[0.507,0.654]
Observations	106

95% confidence intervals in brackets
Values are regression coefficients from fractional GLM with logit link
* $p < 0.05$, ** $p < 0.01$, *** $p < 0.001$

	M63
DParaFovea	
Cholesterol (mg/dl)	0.000
	[-0.001,0.001]
T1DM	0.000
	[0.000,0.000]
Constant	0.545***
	[0.396,0.694]

Observations	106

95% confidence intervals in brackets
Values are regression coefficients from fractional GLM with logit link
* $p < 0.05$, ** $p < 0.01$, *** $p < 0.001$

	M64
DParaFovea	
Triglycerides (mg/dl)	-0.000
	[-0.001,0.001]
T1DM	0.000
	[0.000,0.000]
Constant	0.585***
	[0.546,0.624]
Observations	106

95% confidence intervals in brackets
Values are regression coefficients from fractional GLM with logit link
* $p < 0.05$, ** $p < 0.01$, *** $p < 0.001$

	M65
DTemporal	
Age (years)	-0.004
	[-0.007,0.000]
T1DM	-0.057***
	[-0.087,-0.027]
Constant	0.658***
	[0.599,0.718]
Observations	202

95% confidence intervals in brackets
Values are regression coefficients from fractional GLM with logit link
* $p < 0.05$, ** $p < 0.01$, *** $p < 0.001$

	M66
DTemporal	
T1DM duration	-0.003
(years)	[-0.009,0.003]
T1DM	0.000
	[0.000,0.000]
Constant	0.567***
	[0.522,0.612]
Observations	106

95% confidence intervals in brackets
Values are regression coefficients from fractional GLM with logit link
* $p < 0.05$, ** $p < 0.01$, *** $p < 0.001$

	M67
DTemporal	
Age at onset (years)	0.002

	[-0.004,0.009]
T1DM	0.000
	[0.000,0.000]
Constant	0.526***
	[0.467,0.586]
Observations	106

95% confidence intervals in brackets
Values are regression coefficients from fractional GLM with logit link
* $p < 0.05$, ** $p < 0.01$, *** $p < 0.001$

	M68
DTemporal	
Mean HbA1c from	-0.029*
onset to OCT (%)	[-0.052,-0.005]
T1DM	0.000
	[0.000,0.000]
Constant	0.769***
	[0.589,0.949]
Observations	100

95% confidence intervals in brackets
Values are regression coefficients from fractional GLM with logit link
* $p < 0.05$, ** $p < 0.01$, *** $p < 0.001$

	M69
DTemporal	
LDL-cholesterol	-0.000
(mg/dl)	[-0.001,0.001]
T1DM	0.000
	[0.000,0.000]
Constant	0.571***
	[0.455,0.686]
Observations	106

95% confidence intervals in brackets
Values are regression coefficients from fractional GLM with logit link
* $p < 0.05$, ** $p < 0.01$, *** $p < 0.001$

	M70
DTemporal	
HDL-cholesterol	0.000
(mg/dl)	[-0.002,0.002]
T1DM	0.000
	[0.000,0.000]
Constant	0.526***
	[0.421,0.632]

Observations	106

95% confidence intervals in brackets
Values are regression coefficients from fractional GLM with logit link
* $p < 0.05$, ** $p < 0.01$, *** $p < 0.001$

	M71
DTemporal	
Cholesterol (mg/dl)	-0.000
	[-0.001,0.001]
T1DM	0.000
	[0.000,0.000]
Constant	0.574***
	[0.409,0.739]
Observations	106

95% confidence intervals in brackets
Values are regression coefficients from fractional GLM with logit link
* $p < 0.05$, ** $p < 0.01$, *** $p < 0.001$

	M72
DTemporal	
Triglycerides (mg/dl)	-0.000
	[-0.001,0.001]
T1DM	0.000
	[0.000,0.000]
Constant	0.566***
	[0.512,0.620]
Observations	106

95% confidence intervals in brackets
Values are regression coefficients from fractional GLM with logit link
* $p < 0.05$, ** $p < 0.01$, *** $p < 0.001$

	M73
DSuperior	
Age (years)	-0.002
	[-0.006,0.002]
T1DM	-0.004
	[-0.038,0.029]
Constant	0.672***
	[0.616,0.729]
Observations	202

95% confidence intervals in brackets
Values are regression coefficients from fractional GLM with logit link
* $p < 0.05$, ** $p < 0.01$, *** $p < 0.001$

	M74
DSuperior	
T1DM duration	-0.002

(years)	[-0.006,0.003]
T1DM	0.000
	[0.000,0.000]
Constant	0.650***
	[0.610,0.691]
Observations	106

95% confidence intervals in brackets
Values are regression coefficients from fractional GLM with logit link
* $p < 0.05$, ** $p < 0.01$, *** $p < 0.001$

	M75
DSuperior	
Age at onset (years)	0.001
	[-0.004,0.007]
T1DM	0.000
	[0.000,0.000]
Constant	0.628***
	[0.570,0.686]
Observations	106

95% confidence intervals in brackets
Values are regression coefficients from fractional GLM with logit link
* $p < 0.05$, ** $p < 0.01$, *** $p < 0.001$
